# Exploring predictors of aggressive intrusive thoughts and aggressive scripts: Similarities and differences in phenomenology

**DOI:** 10.1002/ab.22061

**Published:** 2022-11-21

**Authors:** Stephanie J. Fernandez, Michael Daffern, Richard Moulding, Maja Nedeljkovic

**Affiliations:** ^1^ Department of Psychological Sciences Swinburne University of Technology Hawthorn Melbourne Australia; ^2^ Centre for Forensic Behavioural Science Swinburne University of Technology Alphington Melbourne Australia; ^3^ The Cairnmillar Institute Camberwell Melbourne Australia

**Keywords:** aggressive intrusive thoughts, aggressive behavior, aggressive scripts, ego‐dystonicity, obsessive beliefs

## Abstract

Experiencing a thought about harming or injuring another person is commonly reported by the general population. Aggressive intrusive thoughts (AITs) and aggressive scripts are two constructs commonly used to define the experience of thinking about harming another person. However, they are generally investigated separately and with two significantly different population groups; respectively, individuals with obsessive‐compulsive disorder and people with a history of violent behavior. AITs and aggressive scripts are assumed to have very different implications for violence risk assessment, but conceptual overlap and an absence of empirical research renders this assumption premature. Using a battery of self‐report measures, this study aimed to investigate the differential predictors of AITs and aggressive script rehearsal in a nonclinical sample. Additionally, using regression analyses, the predictors of self‐reported aggressive behavior were explored in a sample of 412 adults (73% females; *M*_age_ = 31.96 years, SD = 11.02). Violence‐supportive beliefs and frequency of anger rumination predicted the frequency of aggressive script rehearsal, and aggressive script rehearsal, anger rumination, and violence‐supportive beliefs predicted a history of aggressive behavior. In contrast, obsessive beliefs were predictive of AITs, and only AITs were related to ego‐dystonicity. Both AITs and aggressive script rehearsal were related to the use of thought control strategies. These findings support the contributions that maladaptive beliefs have in the experience of aggressive scripts and AITs. Beliefs about violence, a history of aggressive behavior, and ego‐dystonicity appear to differentiate aggressive scripts from AITs.

## 1 INTRODUCTION

The outcomes associated with thoughts about inflicting harm or injury to others differ significantly depending on the population group that experience these thoughts. In some instances, aggressive thoughts are associated with aggressive acts and violence (Gilbert & Daffern, 2017; Grisso et al., 2000), and in other cases, individuals will go to significant lengths to prevent harm occurring to others (Pascual‐Vera et al., 2019; Rowa & Purdon, 2003). In particular, in people diagnosed with obsessive‐compulsive disorder (OCD), aggressive intrusive thoughts (AITs) are purportedly not associated with subsequent acts of aggression (Veale et al., 2009), whereas in offender populations thoughts with similar content, typically referred to as aggressive scripts, are associated with aggressive behavior^1^ (Daff et al., 2015). Historically, these phenomena have been investigated separately and within these two diverse population groups, and the associated features of these two constructs have not been compared, conceptually or empirically. Such an examination is overdue for improving risk assessment and intervention by clinicians.

AITs are a common feature of OCD, with approximately 58% experiencing aggressive obsessions as one of their main symptoms (*N* = 485; Pinto et al., 2008); however, the unwanted, distressing, and ego‐dystonic (i.e., contradict an individual's sense of self) features of these thoughts are said to protect against acts of violence (Veale et al., 2009). Rather, AITs induce significant fear and apprehension in those who experience them, influencing compulsive behaviors that reduce one's distress and avert the perceived consequences (e.g., ensuring loved ones are safe and not at risk of harm; Rachman, 1997; Veale et al., 2009). Contrastingly, aggressive scripts are defined as thoughts or daydreams about physically harming or injuring another person and they are used to guide behavior and to regulate emotions (Hosie et al., 2021). When studied in violent offender samples, aggressive script rehearsal have been shown to relate to aggressive behavior; specifically, the more one rehearses their aggressive scripts in mind, the more likely they are to act aggressively (Gilbert & Daffern, 2017).

### 1.1 Cognitive models of AITs and aggressive scripts

According to the cognitive‐behavioral model of OCD, AITs are interpreted through beliefs that cause these thoughts to be viewed as abhorrent, dangerous, or threatening (Moulding et al., 2011; Rachman, 1997; Radomsky et al., 2014). These beliefs include thought action fusion—that a thought about harming another person is equivalent to the imagined action (e.g., “thinking about hurting my loved ones is the same as actually hurting them”; Shafran & Rachman, 2004), and feared self‐beliefs, where the individual believes they possess bad, dangerous, or immoral characteristics as a result of experiencing unwanted thoughts (e.g., “I must be a dangerous person for thinking about harming another person”; Aardema & O'Connor, 2007; Jaeger et al., 2021; Moulding et al., 2011; Shafran & Rachman, 2004). Both Veale et al. (2009) and Fairbrother et al. (2022) suggest that there should be no concern regarding whether a person with OCD will carry out their aggressive intrusions, as they are highly ego‐dystonic to the individual and are associated with significant distress and trepidation.

Research exploring aggressive script rehearsal is based upon social‐cognitive developmental models, like the General Aggression Model and Script Theory, which suggest that once aggressive scripts are created, either by observing aggression or acting aggressively, they are maintained through positive beliefs about aggression (Bushman & Anderson, 2002; Huesmann, 1998). These beliefs include attitudes that endorse aggressive behavior (e.g., “Sometimes you have to fight to keep your self‐respect”; Mills et al., 2002; p. 249), and these beliefs have been found to associate with aggressive scripts rehearsal and aggressive behavior, and are common in some offender populations (Gilbert & Daffern, 2017; Huesmann, 1988; Kelty et al., 2011). Further, alternative cognitive models, such as the Multiple Systems Model (Denson (2013), propose that understanding how and why individuals engage in “angry rumination” (a related but not synonymous construct; Hosie et al., 2022) may identify the precipitants of aggressive behavior (Denson, 2013). As emphasized by Hosie et al. (2022), anger rumination is concerned with perseverative thinking over experiences of anger, which may also include ruminations regarding past provocations. However, Hosie et al. (2022) argue that anger rumination that includes thoughts of retaliation (i.e., one preparedness and plans for revenge) is synonymous with some aggressive scripts (although some other aggressive scripts are rehearsed outside of the context of angry rumination, for instance, in a more pleasurable planful state).

### 1.2 The appraisal process of AITs and aggressive scripts

#### 1.2.1 Aggressive intrusive thoughts

Current understandings of AITs in OCD highlight how beliefs underly the appraisal of these intrusions (Rowa & Purdon, 2003). For example, Rowa and Purdon (2003) found that AITs are associated with beliefs of responsibility and the need to control thoughts (*N* = 64). When explained in the context of experiencing AITs, these beliefs: (1) influence the preoccupation with one's responsibility over the content of the thought (e.g., “I must ensure my loved ones are safe from harm”), and (2) influence one to believe they should control their thoughts (e.g., “I must not have thoughts with abhorrent contents and should control my thoughts”). These forms of appraisal have been found to promote neutralizing or thought control behaviors (e.g., distraction strategies; or checking) that are dysfunctional, and that in turn maintain the experience of OC symptoms like AITs (Amir et al., 1997; Jacoby et al., 2015).

Within OCD, the content of the intrusive thought is experienced as ego‐dystonic to the individual, and the initial appraisal of the thought includes a sense of disbelief about thinking unpleasant or unacceptable content (Lee & Kwon, 2003; Purdon et al., 2007). Purdon et al. (2007) found that in an OCD sample, experiencing thoughts classed as repugnant was related to these thoughts being experienced as ego‐dystonic and inconsistent with one's morals. Purdon et al. (2007) also identified that intrusive thoughts initially appraised as ego‐dystonic can, over time, become accommodated into one's self‐concept and be reappraised as ego‐syntonic. Similarly, Bhar and Kyrios (2007) identified that individuals who demonstrate ambivalence about their sense of self, morality, or lovability (e.g., “I question whether I am a moral person”; Bhar & Kyrios, 2007; p. 1855) are likely to experience obsessional thoughts and behaviors. Beliefs concerning self‐perceptions, including the feared self, have been proposed to influence the appraisal and reoccurrence of AITs. It is highlighted that individuals with feared self‐beliefs are likely to perceive themselves as “immoral”, “dangerous,” or “insane” for experiencing thoughts with unacceptable contents (Aardema et al., 2017; Ferrier & Brewin, 2005).

#### 1.2.2 Aggressive scripts

Beliefs related to aggressive script rehearsal are centered on the acceptability of the imagined aggressive behaviors (Gilbert & Daffern, 2017). In a nonclinical sample of students and community participants, Kelty et al. (2011) found that individuals who endorse violent and aggressive beliefs were likely to rehearse aggressive scripts and engage in aggressive behavior. The maintenance of aggressive scripts is also moderated by one's past experiences with aggression and violence, suggesting that a person's life history with aggressive behaviors (either through observation or direct experiences) is highly predictive of a person's aggressive tendencies (Gilbert & Daffern, 2017).

Fewer studies have focused on the role an individual's subjective experience has in aggressive script rehearsal and their appraisal. Hosie et al. (2021) investigated the emotional sequelae associated with aggressive script rehearsal in a sample of incarcerated offenders (*N* = 131). The most common emotion associated with aggressive script rehearsal was anger, followed by hate, fear, sadness, confusion, and annoyance. It was also identified that offenders with a greater history of aggressive behavior were more likely to experience feelings of excitement when rehearsing aggressive scripts, when compared with offenders with less significant histories of aggression. These findings highlight variability in the emotional reactions associated with aggressive script rehearsal, and also the relationship between aggressive script rehearsal and aggressive behavior.

### 1.3 Neutralization and thought control strategies

There is limited research that has examined the thought control methods employed to manage AITs or aggressive scripts, specifically. Studies of OCD have found that a range of control strategies can be employed to manage unacceptable intrusive thoughts, such as AITs, including self‐punishment, avoidance, and seeking reassurance (Belloch et al., 2004; Jacoby et al., 2015; Lee & Kwon, 2003). However, the effectiveness of these control strategies has been questioned, as research suggests that certain methods (e.g., self‐punishment) can increase the severity and frequency of intrusive thoughts (Jacoby et al., 2015). With regard to aggressive scripts, Nagtegaal et al. (2006) found that in an undergraduate student sample (*N* = 72), in which 60% reported rehearsal of aggressive scripts, distraction and reappraisal techniques were the most common thought control method utilized. Nagtegaal et al. (2006) also found that self‐punishment techniques were associated with hostility and aggression. These findings across intrusive thought and aggressive script research highlight that different thought control strategies may be utilized to manage different thought experiences. However, the research also indicates that certain methods may have negative implications on the thought experience, and one's affect.

### 1.4 Current study

To our knowledge, no study has concurrently examined the relationships between cognitive predictors of AITs and aggressive script rehearsal, nor have features of these phenomena been empirically investigated in the same study. As such, in the present study, cognitive predictors related to OCD symptoms, and beliefs related to violent attitudes and aggressive behavior, were investigated. The contributions of the feared self, ego‐dystonicity, and thought control strategies were also examined. It was hypothesized that ego‐dystonicity, self‐ambivalence, and the fear of self would predict AITs. The association between thought control strategies with AITs and aggressive scripts was explored. It was hypothesized that violent attitudes, anger rumination, and a history of aggressive behavior would predict aggressive script rehearsal. It was hypothesized that general OCD beliefs and thought control strategies would predict the experience of OC symptoms. It was also hypothesized that anger rumination and violent attitudes would predict past aggressive behavior. Given the relationship that exists between OCD and depressive symptoms, and the influence that depression and anxiety can have on aggressive thoughts (Ching et al., 2017), depression, anxiety, and stress were controlled for the in the present study.

## 2 METHODS

### 2.1 Participants

The original sample comprised 460 participants, and after validity checks were conducted, the final sample included 412 English‐speaking nonclinical subjects aged between 18 and 69 (*M*_age_ = 31.96; SD = 11.02; 73% females) who did not fail attentional control questions and completed all questionnaires. Majority of participants resided in Australia (*n* = 401), with the remaining in other countries (*n* = 9). Two participants did not disclose where they were residing. Review of clinical measures (i.e., Depression, Anxiety Stress Scale—21; OC Inventory‐Revised) indicated that approximately 31% of participants experienced moderate to severe levels of stress symptoms. Depression, anxiety, and OC symptoms were within normal range.

Participants were recruited using an undergraduate psychology research experience program from an Australian University. Students participated in the study in exchange for course credit. Participants were also recruited through advertisements on social media (Gumtree, Whirlpool, Twitter, and Reddit), and additional participants were obtained via “snowball” methods initiated by those who participated. Participants recruited via social media streams were offered the opportunity to enter into a draw for one of four AUD$100 gift vouchers.

### 2.2 Measures

#### 2.2.1 Anger rumination scale (ARS): Thoughts of revenge subscale

The present study only utilized the Thoughts of Revenge subscale of the ARS, which measures thoughts about anger and retribution after provoking situations (Sukhodolsky et al., 2001). Participants rate items such as “I have long‐living fantasies of revenge after the conflict is over” on a 4‐point Likert scale ranging from 1 (almost never) to 4 (almost always). The original ARS has demonstrated adequate internal consistency (*α*_s_ = .72–.86) and good test‐retest reliability (*r* = .77) for a 1‐month period (Sukhodolsky et al., 2001). In the current study, the Thoughts of Revenge subscale demonstrated good internal consistency (*α* = .75).

#### 2.2.2 Depression anxiety stress scale (DASS) short form

The DASS is a 21‐item self‐report measure that assesses emotional states of depression, anxiety, and stress symptoms, and comprises three subscales; Depression, Anxiety, and Stress (Lovibond & Lovibond, 1995). Participants rate items such as “I felt that life was meaningless” with reference to the past week, on a 4‐point Likert scale ranging from 0 (did not apply to me at all) to 3 (applied to me very much or most of the time). In the current study, the DASS‐21 demonstrated excellent internal consistency (*α* = .94), and good internal consistency across the three subscales (*α*_s_= .86–.92).

#### 2.2.3 Ego‐dystonicity questionnaire (EDQ)‐reduced version

The EDQ is a 27‐item self‐report measure that assesses the extent to which one believes the content of their thoughts is inconsistent with their self‐beliefs, values, and moral attitude (Belloch et al., 2012). For the present study, the EDQ was modified to ask participants to focus on their most upsetting “AITs” whilst providing their ratings. Participants rate items such as that the “Thought is immoral” on a 7‐point Likert scale ranging from 1 (strongly agree) to 7 (strongly disagree). The EDQ‐reduced version demonstrated good internal consistency in the present study (*α*_s_ = .94).

#### 2.2.4 Fear of self questionnaire (FSQ)

The FSQ is an 8‐item self‐report measure that assess beliefs pertaining to covert aspects of one's personality (Aardema et al., 2013). Participants rate items such as “I often question my own character” on a 6‐point Likert scale ranging from 1 (strongly disagree) to 6 (strongly disagree). In the current study the FSQ‐8 demonstrated good internal consistency (*α* = .88).

#### 2.2.5 Life history of aggression (LHA)

The LHA, as revised by Coccaro et al. (1997), is a self‐report measure that assesses the number of occurrences of aggressive behaviors since the age of 13. The present study only utilized the Aggression subscale which measures overt experiences of aggressive behavior. Participants rate items such as “Temper Tantrums” on a 5‐point Likert scale ranging from 0 (no occurrences) to 5 (more events than can be counted). In the current study, the Aggression subscale demonstrated good internal consistency (*α* = .79).

#### 2.2.6 Measures of criminal attitudes and associations (MCAA)

The complete MCAA consists of a two‐part self‐report questionnaire, comprising Violence, Entitlement, Antisocial Intent, and Associates subscale (Mills et al., 2002). For the present study only 13 items pertaining to the Violence subscale were used, where items such as “There is nothing wrong with beating up someone who asks for it” are rated on a dichotomous scale of agree/disagree. In the current study, the subscale of Violence demonstrated good internal consistency (*α* = .81).

#### 2.2.7 Obsessive beliefs questionnaire (OBQ‐20)

The OBQ‐20 is a short form of the Obsessive Beliefs Questionnaire (Obsessive Compulsive Cognitions Working Group & OCCWG, 2005) (Moulding et al., 2011). A 20‐item self‐report measure, the OBQ assesses four obsessive beliefs identified through factor analyses: (1) Threat, (2) Responsibility, (3) Importance of Thoughts, and (4) Perfectionism. Participants rate items such as “I should be upset if I make a mistake” on a 7‐point Likert scale ranging from 1 (disagree very much) to 7 (agree very much). In the current study, the OBQ‐20 demonstrated excellent internal consistency (*α* = .91), and good internal consistency across the four subscales (*α*_s_ = 0.77–0.85).

#### 2.2.8 **Obsessive‐**compulsive inventory‐revised (OCI‐R)

The OCI‐R is an 18‐item self‐report measure that assesses OC symptoms and associated distress associOCI‐Rated. Participants rate items such as “I find it difficult to control my own thoughts” on a 4‐point Likert scale ranging from 0 (not at all) to 4 (extremely) (Foa et al., 2002). In the current study, the OCI‐R demonstrated good internal consistency across the six subscales (*α*_s_ = .65–.87).

#### 2.2.9 Schedule of imagined violence (SIV)

The original SIV is a set of eight items that explore details relating to participants' experience of a violent thought, including subsequent aggressive actions (Grisso et al., 2000). The current study only utilized the frequency item of the SIV (“How often do you have thoughts about hurting or injuring other people?”). Participants rated their responses to this item on a 7‐point Likert scale ranging from 0 (never) to 7 (several times a day). This item has been used in prior research to measure the frequency of one's aggressive script rehearsal (Daff et al., 2015; Hosie et al., 2022; Podubinski et al., 2017).

#### 2.2.10 Self‐ambivalence measure (SAM)

The SAM is a 19‐item measure of self‐ambivalence—which encompasses beliefs regarding uncertainty toward the self, and dichotomous perceptions about one's self‐concept (Bhar & Kyrios, 2007). Participants respond to items such as “I have mixed feelings about my self‐worth” on a 5‐point Likert scale ranging from 0 (not at all) to 4 (agree totally). In the current study, the total scale of the SAM was used, and it demonstrated excellent internal consistency (*α* = .93).

#### 2.2.11 Thought control questionnaire (TCQ)

A recent psychometric study conducted by Luciano et al. (2006) confirmed a five‐factor model of the TCQ with 16 items, which the current study utilized (Luciano et al., 2006). Participants rate items such as “I punish myself for thinking the thought” on a 4‐point Likert scale ranging from 1 (never) to 4 (almost always). The 16‐item version of the TCQ demonstrated adequate internal consistency in the current study (*α* = .67).

#### 2.2.12 Questionnaire of unpleasant intrusive thoughts (QUIT)

Derived from earlier measures of intrusive thoughts (Clark et al., 2014; García‐Soriano et al., 2011; Purdon & Clark, 1993), the QUIT assesses the experience of specific themes of intrusive thoughts (Pascual‐Vera et al., 2019). Only the unpleasant content domain was used in the current study, and analyses involving the QUIT only used the frequency item of unwanted AITs. Participants rated their responses on a 7‐point Likert scale ranging from 0 (never) to 6 (always).

### 2.3 Procedure

After participants provided consent, the online questionnaire was administered with responses recorded anonymously via Qualtrics. After completing demographic questions (e.g., age, gender, place of residence, education level) the measures were presented in random order.

## 3 RESULTS

### 3.1 Data inspection

Data analysis was conducted using IBM SPSS Statistics 27 for PC. Preliminary data inspection revealed that five items were missing from the survey across three variables (one item from OBQ‐20; two items from MCAA, and two items from TCQ) due to administration error (*n* = 223). The survey was amended to include all missing items, and the amended survey was used in all subsequent data collection. Once data collection was completed and an additional 237 participant responses were recorded (*N* = 460), an Expectation‐Maximisation (EM) algorithm was applied to the data set to impute the missing item scores from the initial survey. The utilization of an EM algorithm for the missing items was justified as Little's missing completely at random (MCAR) test indicated that the data was MCAR: (OBQ‐20; *χ* ^2^ [68, *N* = 460] = 57.98, *p* = .80; MCAA; *χ* ^2^ [49, *N* = 460] = 532.12, *p* = .97; and TCQ; *χ* ^2^ [68, *N* = 460] = 86.97, *p* = .06). A missing values analysis was performed on the entire data set and this revealed that several scales also contained missed data. Little's MCAR tests were performed on these scales which indicated that data was MCAR, thus missing data was imputed using the EM algorithm. Data inspection identified several scales were significantly skewed, which is a common trend in nonclinical samples. Transformations on skewed scales were performed to normalize the distributions; however, some of the scales remained skewed. The assumptions for regression analyses were met.

### 3.2 Correlational analyses

Pearson's correlations were performed between variables (Table 1). Obsessive‐compulsive symptoms (i.e., OCI‐R scores) were related significantly to general OCD beliefs (OBQ), thought control (TCQ), anger rumination (ARS), history of aggressive behavior (LHA), and violence‐supportive beliefs (MCAA) at a weak‐to‐moderate level, with the strongest correlation with general OCD beliefs. AITs (QUIT) were significantly related to anger rumination, LHA, criminal attitudes, ego‐dystonicity (EDQ), feared self‐beliefs (FSQ), and self‐ambivalence (SAM), at a weak‐to‐moderate level. AITs related at a weak‐to‐moderate level with all OCI‐R symptom dimensions except for the washing dimension. AITs related to all dimensions of obsessive beliefs (OBQ), except for the responsibility, and importance/control of thoughts dimension. AITs related to all dimensions of thought control (TCQ), except for the worry, and social dimensions. Finally, aggressive scripts (SIV) were found to relate in a significant positive direction with a history of aggressive behavior, anger rumination, and violence supportive beliefs at a moderate level. Aggressive scripts correlated with AITs, feared self‐beliefs, and symptom dimensions.

**Table 1:** Pearson's Correlations for all variables

							OBQ				
	ARS	DASS	EDQ	FSQ	LHA	MCAA	TotalOBQ	1. T	2. R	3. I/C	4. P/U
ARS	1										
DASS	.382**	1									
EDQ	.058	.250**	1								
FSQ	.435**	.538**	.233**	1							
LHA	.390**	.235**	.022	.306**	1						
MCAA	.417**	.157**	−.042	.206**	.309**	1					
OBQ											
Total OBQ	.219**	.523**	.358**	.540**	.145**	.085	1				
1. Threat	.362**	.551**	.251**	.629**	.272**	.171**	.796**	1			
2. Responsibility	.065	.328**	.349**	.270**	.091	−.050	.746**	.433**	1		
3. I/C	.034	.335**	.277**	.352**	−.027	.017	.756**	.478**	.416**	1	
4. P/U	.225**	.438**	.251**	.458**	.123*	.133**	.839**	.610**	.489**	.505**	1
OCI											
Total OCI−R	.466**	.605**	.287**	.536**	.247**	.237**	.528**	.542**	.296**	.359**	.469**
1. Washing	.156**	.275**	.136**	.270**	.101*	.129**	.382**	.333**	.274**	.304**	.304**
2. Obsessing	.433**	.654**	.201**	.548**	.271**	.207**	.464**	.528**	.256**	.308**	.382**
3. Hoarding	.301**	.251**	.194**	.294**	.193**	.178**	.234**	.246**	.121*	.165**	.208**
4. Checking	.267**	.339**	.176**	.323**	.086	.166**	.338**	.357**	.154**	.294**	.268**
5. Ordering	.393**	.518**	.253**	.422**	.232**	.159**	.470**	.434**	.285**	.278**	.475**
6. Neutralising	.254**	.307**	.168**	.335**	.101*	.176**	.358**	.356**	.171**	.276**	.327**
SAM	.376**	.636**	.275**	.753**	.259**	.130**	.638**	.669**	.355**	.415**	.571**
TCQ											
Total TCQ	.017	.151**	.187**	.142**	.056	.004	.213**	.170**	.195**	.218**	.102*
1. Distract	−.129**	−.154**	.128**	−.182**	−.046	−.023	−.031	−.059	.022	.032	−.076
2. Punish	.176**	.389**	.381**	.417**	.132**	.051	.414**	.344**	.246**	.352**	.356**
3. Worry	.207**	.423**	.132**	.332**	.201**	.095	.302**	.304**	.177**	.229**	.241**
4. Reappraise	−.092	−.029	−.023	−.021	−.060	−.150**	.004	−.021	.103*	−.030	−.067
5. Social	.019	.040	−.030	.078	.044	.086	.077	.081	.071	.138**	−.018
QUIT	.310**	.280**	.165**	.282**	.142**	.204**	.125*	.258**	−.021	.007	.144**
SIV	.485**	.246**	.021	.326**	.426**	.374**	.130**	.272**	.037	−.044	.146**
Mean	.813	5.53	126.55	25.90	8.90	14.46	69.51	15.98	21.83	3.48	18.78
Std.	.135	2.27	29.35	10.88	5.69	2.41	21.22	6.34	6.73	.888	7.35
Min	.600	0	27	8	0	12	19.71	5	5	2.24	4.71
Max	1.20	10.95	189	53	23	23.25	131.02	34	35	5.83	35

	OCI−R							
	TotalOCI	1. W	2. Ob	3. H	4. C	5. Or	6. N	SAM
ARS								
DASS								
EDQ								
FSQ								
LHA								
MCAA								
OBQ								
Total OBQ								
1. Threat								
2. Responsibility								
3. I/C								
4. P/U								
OCI								
Total OCI−R	1							
1. Washing	.477**	1						
2. Obsessing	.783**	.310**	1					
3. Hoarding	.659**	.293**	.346**	1				
4. Checking	.683**	.428**	.428**	.327**	1			
5. Ordering	.772**	.380**	.615**	.333**	.419**	1		
6. Neutralising	.613**	.326**	.350**	.353**	.356**	.359**	1	
SAM	.552**	.253**	.569**	.269**	.286**	.467**	.323**	1
TCQ								
Total TCQ	.219**	.247**	.181**	.112*	.183**	.162**	.183**	.190**
1. Distract	.002	.124*	−.048	.026	.053	.007	.031	−.153**
2. Punish	.403**	.218**	.410**	.183**	.241**	.325**	.270**	.481**
3. Worry	.339**	.156**	.359**	.191**	.218**	.270**	.166**	.408**
4. Reappraise	−.056	.085	−.076	−.042	−.026	−.041	−.008	−.01
5. Social	.104*	.167**	.064	.031	.116*	.032	.132**	.06
QUIT	.302**	.050	.329**	.189**	.112*	.258**	.209**	.281**
SIV	.333**	.070	.351**	.242**	.124*	.261**	.213**	.249**
Mean	3.57	1.24	3.79	1.55	1.18	1.72	.854	40.59
Std.	1.23	.913	2.74	.883	.916	.773	.867	16.46
Min	0	0	0	0	0	0	0	4
Max	7.07	3.32	12	3.46	3.46	3.46	3.32	73

	TCQ							
	TotalTCQ	1. D	2. P	3. W	4. R	5. S	QUIT_F	SIV_F
ARS								
DASS								
EDQ								
FSQ								
LHA								
MCAA								
OBQ								
Total OBQ								
1. Threat								
2. Responsibility								
3. I/C								
4. P/U								
OCI								
Total OCI−R								
1. Washing								
2. Obsessing								
3. Hoarding								
4. Checking								
5. Ordering								
6. Neutralising								
SAM								
TCQ								
Total TCQ	1							
1. Distract	.623**	1						
2. Punish	.450**	−.017	1					
3. Worry	.447**	.051	.367**	1				
4. Reappraise	.667**	.293**	.045	.061	1			
5. Social	.600**	.126*	.127*	.137**	.340**	1		
QUIT	−.060	−.103*	.114*	.054	−.119*	−.048	1	
SIV	−.073	−.077	.112*	.079	−.147**	−.092	.377**	1
Mean	33.65	9.94	.635	3.93	7.27	2.80	.660	1.68
Std.	5.26	2.40	.143	1.29	2.06	.303	1.07	1.13
Min	19.30	4	.480	2	3	2	0	1
Max	51.85	16	1.08	8	12	4	5	7

*Note*: N = 410.

Abbreviations: ARS = Anger Rumination Scale; DASS = Depression Anxiety Stress Scale; EDQ = Ego Dystonicity Questionnaire; FSQ = Fear of Self Questionnaire; LHA = Life History of Aggression; MCAA = Measure of Criminal Attitudes and Associates; OBQ = Obsessive Beliefs Questionnaire; OCI‐R = Obsessive Compulsive Inventory‐Revised; SAM = Self Ambivalence Measure; SIV = Schedule of Imagined Violence ‐ Frequency; TCQ = Thought Control Questionnaire; QUIT = Questionnaire of Unpleasant Intrusive Thoughts ‐ Frequency.

* *p*<0.05

** *p*<0.01

*** *p*<0.001.

of the OCI‐R except the washing dimension, at a weak‐to‐moderate level. All dimensions of the OBQ related to aggressive scripts at a weak‐to‐moderate level, except the responsibility, and importance/control of thoughts dimension. Aggressive scripts related to all dimensions of thought control, except for the distraction, worry, and social dimensions.

### 3.3 Regression analyses

Regression analyses are presented in Table 2. Depression, anxiety, and stress were entered as a total at Stage 1, and all other variables at Stage 2. Regression analyses examined the predictors of AITs (QUIT) and aggressive script rehearsal (SIV). The regression analysis also examined the predictors of LHA, and OC symptoms. The assumptions for regression analyses were met.

**Table 2:** Beta coefficients (*t* statistics) for regression analyses predicting AITs, aggressive scripts (SIV), obsessive‐compulsive symptoms (OCI), and life history of aggression (LHA)

	QUIT		SIV		OCI‐R		LHA	
	Step 1	Step 2	Step 1	Step 2	Step 1	Step 2	Step 1	Step 2
DASS	0.28 (5.89***)	0.089 (1.42)	0.253 (5.27***)	−0.030 (−0.53)	0.607 (15.44***)	0.296 (6.30***)	0.232 (4.81***)	0.051 (0.82)
ARS		0.059 (1.01)		0.233 (4.54***)		0.187 (4.16***)		0.154 (2.67**)
EDQ		0.126 (2.63*)		−0.02 (−0.45)		0.077 (2.03*)		−0.026 (−0.54)
FSQ		0.029 (0.41)		0.107 (1.68)		0.098 (1.77)		0.092 (1.31)
MCCA		0.064 (1.28)		0.120 (2.69**)		0.036 (0.92)		0.137 (2.77**)
OBQ		−0.152 (−2.46*)		−0.038 (−0.69)		0.201 (4.20***)		−0.032 (−0.52)
SAM		0.148 (1.89)		−0.041 (−0.58)		0.009 (0.14)		0.06 (0.77)
TCQ		−0.092 (−1.98*)		−0.101 (−2.44*)		0.108 (2.99**)		0.061 (1.32)
OCI‐R		0.098 (1.53)		0.125 (2.18*)		–		−0.036 (0.57)
LHA		−0.068 (−1.36)		0.214 (4.87***)		−0.023 (−0.57)		–
SIV		0.255 (4.68***)		–		0.095 (2.19*)		0.263 (4.87***)
QUIT		–		0.204 (4.68***)		0.06 (1.54)		−0.067 (−1.36)
*R* ^2^	0.078	0.230	0.064	0.384	0.369	0.530	0.054	0.241
Δ*R* ^2^		.152**		.321***		.161***		.187***

*Note*: *N* = 410. Step 1: DASS; Step 2: ARS, EDQ, FSQ, MCAA – Violence, OBQ, SAM, TCQ, OCI, SIV. Standardized beta coefficients; *t* statistics in parentheses.

Abbreviations: ARS, anger rumination scale; DASS, depression anxiety stress scale; EDQ, ego‐dystonicity questionnaire; FSQ; fear of self questionnaire; LHA, life history of aggression; MCAA, measure of criminal attitudes and associates‐ violence subscale; OBQ, obsessive beliefs questionnaire; OCI‐R, obsessive‐compulsive inventory‐revised; SAM, self ambivalence measure; SIV, schedule of imagined violence–frequency; TCQ, thought control questionnaire; QUIT, questionnaire of unpleasant intrusive thoughts–frequency.

* *p* < .05

** *p* < .01

*** *p* < .001.

Examining the differential predictors of AITs (QUIT), the DASS at stage 1 explained 8% of the variance in QUIT, and the remainder 10 predictors explained an additional 15%, *F* change (10, 398) = 7.85, *p* < .001. In the final model, the SIV, EDQ, OBQ, and TCQ were all significant predictors of QUIT. Examining predictors of aggressive script rehearsal (SIV), the DASS explained 6% of the variance in aggressive scripts, with the additional 10 predictors explaining an additional 32% of the variance in the SIV after controlling for psychological well‐being, *F* change (10, 398) = 20.73, *p* < .001. In the final model the ARS, LHA, QUIT, MCAA, and TCQ were all significant predictors of aggressive script rehearsal. Examination of regression weights identified that the influence of obsessive beliefs on QUIT was negative. This result indicated a suppression effect, as the OBQ is a univariate positive predictor of QUIT frequency. To identify which variables were suppressing the OBQ, repeated regression analyses were conducted where each variable in the model was excluded in turn from the analysis. These analyses indicated that the DASS, OCI‐R, FSQ and SAM were suppressing the OBQ, with obsessional beliefs nonsignificantly associated with QUIT (*β* = .003, *p* > .05) when they were removed from the equation.

Repeating the analysis for OC symptoms, the DASS explained 37% of the variance and a further 16% of the variance was predicted by the other variables, *F* change (10, 398) = 13.60, *p* < .001. In the final model, the DASS, OBQ, ARS, TCQ, SIV, and the EDQ were significant predictors. For a history of aggressive behavior, the DASS explained 5% of the variance, and the additional 10 predictors were entered at stage 2, the total variance explained by the entire model was 24%, *F* (11, 398) = 11.47, *p* < .001. The inclusion of 10 predictors explaining an additional 19%, *F* change (10, 398) = 9.80, *p* < .001. Overall, the SIV, ARS, and the MCAA were significant.

## 4 DISCUSSION

This study investigated the association between different OCD‐relevant beliefs, self‐themes, and aggression‐related beliefs with AITs and aggressive script rehearsal. As hypothesized, general OCD beliefs and thought control strategies predicted OC symptoms. Unexpectedly, anger rumination and the frequency of aggressive script rehearsal also predicted OC symptoms. It was also found, as hypothesized, that ego‐dystonicity predicted the experience of AITs. Contrary to expectations, the feared self was not identified as a unique predictor of AITs or aggressive script rehearsal. As hypothesized, violence supportive beliefs, a history of aggressive behavior, anger rumination, and the use of thought control strategies predicted aggressive script rehearsal. It was also found, as hypothesized, that anger rumination, violence supportive beliefs, and the frequency of aggressive script rehearsal predicted a history of aggressive behavior. No relationship was found between aggressive scripts and ego‐dystonicity. These findings are unpacked in detail below.

First, and consistent with prior research (Moulding et al., 2014; Purdon & Clark, 1994), general OCD beliefs and thought control strategies were found to predict OC symptoms. Cognitive appraisal models of OCD postulate that maladaptive beliefs influence the appraisal of intrusive thought experiences (Radomsky et al., 2014) which motivate the use of compulsive behaviors, inadvertently perpetuating the intrusive thoughts (Belloch et al., 2004; Brakoulias et al., 2014; Purdon & Clark, 1993; Salkovskis, 1985; Wheaton et al., 2010). The current study found significant correlations between the thought control strategies of punishment and worry with OC symptoms, suggesting that certain thought control strategies may be less effective than others at controlling the reoccurrence of symptoms; similar findings have been highlighted by Jacoby et al. (2015) who found that using self‐punishment as a means of controlling intrusive thoughts, was associated with frequent repugnant intrusive thoughts. Contrary to expectations, at a multivariate level, anger rumination and aggressive script rehearsal were also found to predict the experience of OC symptoms. An explanation for this finding may concern the measurement of anger rumination and aggressive script rehearsal, which enquires generally about aggressive thinking and may share similarities to measurements of AIT in OCD. It is possible that current measurements of anger rumination, aggressive script rehearsal, and AITs in OCD are unable to clearly differentiate the phenomena, and thus interrelationships across constructs are being identified.

Second, ego‐dystonicity and self‐themes including the feared self and self‐ambivalence were examined. At a univariate level, ego‐dystonicity, feared self‐beliefs, and self‐ambivalence all were significantly related to the experience of AITs. However, at a multivariate level, ego‐dystonicity was found to be the only self‐related predictor of AITs. This aligns with previous research on ego‐dystonicity (Purdon et al., 2007), where experiencing thoughts of harming another person that do not reflect one's intentions, are likely to be interpreted as abhorrent to the self (Lee & Kwon, 2003). Obsessive beliefs were a positive predictor of AITs only when certain measures (e.g., DASS, OCI‐R, FSQ, and SAM) were removed from the model, with analyses indicating a suppression effect due to these variables. One explanation for this finding is that by controlling for depression, anxiety, and OC symptoms, the relationship between AITs and obsessive beliefs no longer identifies the symptomatic elements of intrusive thought experiences. It is unclear how the self‐measures of FSQ and SAM influenced the suppression effect, however, one may speculate that these measures may contain anxiety‐related elements, and when controlled for, the relationship between AITs and obsessive beliefs is no longer able to identify these elements. As these analyses involved a posthoc exploration it is therefore essential that these findings be confirmed in further studies of AITs and obsessive beliefs.

Third, our results suggest an association between the frequency of aggressive script rehearsal and anger rumination, violence supportive beliefs, a history of aggressive behavior, thought control strategies, AITs, and contrary to expectations, also OC symptoms. No relationship was found between aggressive script rehearsal and ego‐dystonicity. The current findings support the notion that the presence of violence supportive beliefs and prior acts of aggression increase the likelihood of aggressive script rehearsal (Hosie et al., 2022; Kelty et al., 2011). Contrary to expectations, thought control strategies were found to have a negative association with aggressive script rehearsal when considered alongside other predictors. This may suggest that the use of thought control strategies for aggressive scripts may act as a protective factor by distracting or occupying one's thoughts on something other than aggression. This aligns with Nagtegaal et al. (2006) who found that distraction, when used as a control strategy, reduced the likelihood of aggressive behavior in participants examined. Additionally, the association between AITs and aggressive script rehearsal can be explained by similarities in measurement instruments. Both measures of AITs and aggressive script rehearsal ask respondents whether they have ever experienced a thought of harming another person. Although it is asked in different ways in instruments used to measure phenomena thought to be related to OCD and aggressive behavior, the overarching content appears to be the same and extant instruments may not have been designed with consideration given to differences in phenomena (AITs and aggressive scripts) across different populations. This appears to demonstrate a critical issue with the measurement of these constructs, and a refinement of these measurement instruments is required. Based on the findings of this study, AITs and aggressive script rehearsal may be differentiated by a history of aggressive behavior, endorsement of violence supportive beliefs, and ego‐dystonicity. Further clinical explorations of these factors is warranted for the purposes of risk assessment and phenomenological understandings of these constructs.

The lack of association between ego‐dystonicity and aggressive script rehearsal may be explained by the anecdotal assumption that individuals with a history of aggressive behavior or who endorse violence supportive beliefs, may experience aggressive thoughts as ego‐syntonic (i.e., consistent with one's self‐concept). As highlighted by Purdon et al. (2007), thoughts that are initially appraised as ego‐dystonic can over time be accommodated for into one's self concept, leading that thought to be interpreted as ego‐syntonic. It may be that a measure of ego‐syntonicity may be more sensitive toward identifying whether an association exists between one's self concept and the process of aggressive script rehearsal—which was not investigated in the present study.

Fourth, anger rumination, aggressive script rehearsal, and violence supportive beliefs were significant predictors of one's propensity to have acted aggressively in the past. This finding is consistent with prior research which suggests that the more one ruminates on aggressive altercations or acts of revenge, the more likely aggressive behavior becomes a part of one's repertoire (Daff et al., 2015; Denson, 2013). LHA was not predicted by AITs which further confirms the understanding that OCD‐related AITs are not associated with overt acts of violence (Veale et al., 2009).

The present study has several strengths, including its novel approach of concurrently measuring AITs and aggressive scripts, as well as exploring relevant features of these phenomena with the aim to better differentiate these constructs from each other. Since this study is one of the first to examine AITs and aggressive script rehearsal concurrently, it has brought attention to the complex task of differentiating AITs from aggressive script rehearsal, and through this, has identified issues with measurement instruments of these constructs. The present study's findings also suggest there are differences in the phenomenology of these constructs, where it appears AITs and aggressive script rehearsal differ particularly on factors concerning a history of aggressive behavior, endorsement of violence supportive beliefs, and the experience of ego‐dystonicity. These features warrant consideration when assessing violence risk, specifically when determining which elements of aggressive thoughts indicate one's propensity to act aggressively, when compared with others.

Nevertheless, the present study's findings should be considered in light of several limitations. The study utilized a cross‐sectional design with self‐report measures, and a limited sample size which prevents causal assumptions being made regarding the relationships found. Similarly, common method variance introduced with self‐report instruments may have influenced the relationships found, and thus results should be interpreted with caution. The use of a nonclinical sample of participants, though a common practice in OCD research (Abramowitz et al., 2014), influences the severity and extent to which OC symptoms and obsessive beliefs are reported. Although the current study was able to identify differential predictors of AITs and aggressive script rehearsal, it should be acknowledged that a limited number of participants endorsed such cognitions, and thus relationships that consider these phenomena should be interpreted accordingly.

The present study supports the relationship that violence supportive beliefs and a history of aggressive behavior have with aggressive script rehearsal. Whether ego‐syntonicity is an important feature of aggressive script rehearsal remains unclear, so further exploration is required. This study has also demonstrated the potential overlap that exists between current measurements of AITs and aggressive script rehearsal, and further explorations of these phenomena concurrently is warranted, where the refinement of these measures would have implications for the clinical utility of these instruments. This study provides implications for risk assessments, as preliminary features that distinguish AITs from aggressive script rehearsal were identified empirically: a history of aggressive behavior, violence supportive beliefs, and ego‐dystonicity. Further, examining these phenomena with clinical population groups may prove beneficial in understanding how these constructs are experienced and maintained by those who frequently report them.

## CONFLICT OF INTEREST

The authors declare no conflict of interest.

## Data Availability

The data that support the findings of this study are available from the corresponding author upon reasonable request.

## ENDNOTE

1 The authors acknowledge that AITs are symptoms that appear in other psychiatric conditions such as anxiety disorders and psychotics disorders (see: Abramowitz et al., 2003; Grisso et al., 2000). However, for the scope of this study, only AITs relevant to OCD have been included. The rationale for not including other psychiatric conditions alongside OCD, particularly psychotic disorders, was due to differences in the appraisal process involved during aggressive thought experiences (see: Clark, 2005; Link et al., 1999).

## References

[ab22061-bib-0001] Aardema, F. , Moulding, R. , Melli, G. , Radomsky, A. S. , Doron, G. , Audet, J. S. , & Purcell‐Lalonde, M. (2018). The role of feared possible selves in obsessive‐compulsive and related disorders: A comparative analysis of a core cognitive self‐construct in clinical samples. Clinical Psychology & Psychotherapy, 25(1), 19–29. 10.1002/cpp.2121 28791792

[ab22061-bib-0002] Aardema, F. , Moulding, R. , Radomsky, A. S. , Doron, G. , Allamby, J. , & Souki, E. (2013). Fear of self and obsessionality: Development and validation of the Fear of Self Questionnaire. Journal of Obsessive‐Compulsive and Related Disorders, 2(3), 306–315. 10.1016/j.jocrd.2013.05.005

[ab22061-bib-0003] Aardema, F. , & O'Connor, K. (2007). The menace within: Obsessions and the self. Journal of Cognitive Psychotherapy, 21(3), 182–197.

[ab22061-bib-0004] Abramowitz, J. S. , Fabricant, L. E. , Taylor, S. , Deacon, B. J. , McKay, D. , & Storch, E. A. (2014). The relevance of analogue studies for understanding obsessions and compulsions. Clinical Psychology Review, 34(3), 206–217. 10.1016/j.cpr.2014.01.004 24561743

[ab22061-bib-0005] Abramowitz, J. S. , Schwartz, S. A. , & Moore, K. M. (2003). Obsessional thoughts in postpartum females and their partners: Content, severity, and relationship with depression. Journal of Clinical Psychology in Medical Settings, 10(3), 157–164. 10.1023/A:1025454627242

[ab22061-bib-0006] Amir, N. , Cashman, L. , & Foa, E. B. (1997). Strategies of thought control in obsessive‐compulsive disorder. Behaviour Research and Therapy, 35(8), 775–777. 10.1016/S0005-7967(97)00030-2 9256520

[ab22061-bib-0007] Belloch, A. , Morillo, C. , Lucero, M. , Cabedo, E. , & Carrió, C. (2004). Intrusive thoughts in non‐clinical subjects: The role of frequency and unpleasantness on appraisal ratings and control strategies. Clinical Psychology & Psychotherapy, 11(2), 100–110. 10.1002/cpp.397

[ab22061-bib-0008] Belloch, A. , Roncero, M. , & Perpiñá, C. (2012). Ego‐syntonicity and ego‐dystonicity associated with upsetting intrusive cognitions. Journal of Psychopathology and Behavioral Assessment, 34(1), 94–106. 10.1007/s10862-011-9255-4

[ab22061-bib-0009] Bhar, S. S. , & Kyrios, M. (2007). An investigation of self‐ambivalence in obsessive‐compulsive disorder. Behaviour Research and Therapy, 45(8), 1845–1857. 10.1016/j.brat.2007.02.005 17408590

[ab22061-bib-0010] Brakoulias, V. , Starcevic, V. , Berle, D. , Milicevic, D. , Hannan, A. , & Martin, A. (2014). The relationships between obsessive‐compulsive symptom dimensions and cognitions in obsessive‐compulsive disorder. Psychiatric Quarterly, 85(2), 133–142. 10.1007/s11126-013-9278-y 24142072

[ab22061-bib-0011] Bushman, B. J. , & Anderson, C. A. (2002). Violent video games and hostile expectations: A test of the general aggression model. Personality and Social Psychology Bulletin, 28(12), 1679–1686. 10.1177/014616702237649

[ab22061-bib-0012] Ching, T. H. W. , Williams, M. , & Siev, J. (2017). Violent obsessions are associated with suicidality in an OCD analog sample of college students. Cognitive Behaviour Therapy, 46(2), 129–140. 10.1080/16506073.2016.1228084 27659199

[ab22061-bib-0013] Clark, D. A. (2005). Intrusive thoughts in clinical disorders: Theory, research, and treatment. Guilford Press.

[ab22061-bib-0014] Clark, D. A. , Abramowitz, J. , Alcolado, G. M. , Alonso, P. , Belloch, A. , Bouvard, M. , Coles, M. E. , Doron, G. , Fernández‐Álvarez, H. , Garcia‐Soriano, G. , Ghisi, M. , Gomez, B. , Inozu, M. , Moulding, R. , Radomsky, A. S. , Shams, G. , Sica, C. , Simos, G. , & Wong, W. (2014). Part 3. A question of perspective: The association between intrusive thoughts and obsessionality in 11 countries. Journal of Obsessive‐Compulsive and Related Disorders, 3(3), 292–299. 10.1016/j.jocrd.2013.12.006

[ab22061-bib-0015] Coccaro, E. F. , Berman, M. E. , & Kavoussi, R. J. (1997). Assessment of life history of aggression: Development and psychometric characteristics. Psychiatry Research, 73(3), 147–157. 10.1016/S0165-1781(97)00119-4 9481806

[ab22061-bib-0016] Daff, E. S. , Gilbert, F. , & Daffern, M. (2015). The relationship between anger and aggressive script rehearsal in an offender population. Psychiatry, Psychology and Law, 22(5), 731–739. 10.1080/13218719.2014.986837

[ab22061-bib-0017] Denson, T. F. (2013). The multiple systems model of angry rumination. Personality and Social Psychology Review, 17(2), 103–123. 10.1177/1088868312467086 23175519

[ab22061-bib-0018] Fairbrother, N. , Collardeau, F. , Woody, S. R. , Wolfe, D. A. , & Fawcett, J. M. (2022). Postpartum thoughts of infant‐related harm and obsessive‐compulsive disorder: Relation to maternal physical aggression toward the infant. The Journal of Clinical Psychiatry, 83(2):39944. 10.4088/JCP.21m14006 35235718

[ab22061-bib-0019] Ferrier, S. , & Brewin, C. R. (2005). Feared identity and obsessive‐compulsive disorder. Behaviour Research and Therapy, 43(10), 1363–1374. 10.1016/j.brat.2004.10.005 16086986

[ab22061-bib-0020] Foa, E. B. , Huppert, J. D. , Leiberg, S. , Langner, R. , Kichic, R. , Hajcak, G. , & Salkovskis, P. M. (2002). The Obsessive‐Compulsive inventory: Development and validation of a short version. Psychological Assessment, 14(4), 485–496. 10.1037/1040-3590.14.4.485 12501574

[ab22061-bib-0021] García‐Soriano, G. , Belloch, A. , Morillo, C. , & Clark, D. A. (2011). Symptom dimensions in obsessive‐compulsive disorder: From normal cognitive intrusions to clinical obsessions. Journal of Anxiety Disorders, 25(4), 474–482. 10.1016/j.janxdis.2010.11.012 21163617

[ab22061-bib-0022] Gilbert, F. , & Daffern, M. (2017). Aggressive scripts, violent fantasy and violent behavior: A conceptual clarification and review. Aggression and Violent Behavior, 36, 98–107. 10.1016/j.avb.2017.05.001

[ab22061-bib-0023] Grisso, T. , Davis, J. , Vesselinov, R. , Appelbaum, P. S. , & Monahan, J. (2000). Violent thoughts and violent behavior following hospitalization for mental disorder. Journal of Consulting and Clinical Psychology, 68(3), 388–398. 10.1037/0022-006x.68.3.388 10883555

[ab22061-bib-0024] Hosie, J. , Dunne, A. , Simpson, K. , & Daffern, M. (2021). Aggressive script rehearsal in adult offenders: Characteristics and association with self‐reported aggression. Journal of Interpersonal Violence, 37, NP21902–NP21926. 10.1177/08862605211062992 34961425

[ab22061-bib-0025] Hosie, J. , Simpson, K. , Dunne, A. , & Daffern, M. (2022). A study of the relationships between rumination, anger rumination, aggressive script rehearsal, and aggressive behavior in a sample of incarcerated adult males. Journal of Clinical Psychology, 78, 1925–1939. 10.1002/jclp.23341 35263441 PMC9541888

[ab22061-bib-0026] Huesmann, L. R. (1998). The role of social information processing and cognitive schema in the acquisition and maintenance of habitual aggressive behavior. Human aggression (pp. 73–109). Academic Press. 10.1016/B978-012278805-5/50005-5

[ab22061-bib-0027] Jacoby, R. J. , Leonard, R. C. , Riemann, B. C. , & Abramowitz, J. S. (2015). Self‐punishment as a maladaptive thought control strategy mediates the relationship between beliefs about thoughts and repugnant obsessions. Cognitive Therapy and Research, 40(2), 179–187. 10.1007/s10608-015-9741-1

[ab22061-bib-0028] Jaeger, T. , Moulding, R. , Yang, Y. H. , David, J. , Knight, T. , & Norberg, M. M. (2021). A systematic review of obsessive‐compulsive disorder and self: Self‐esteem, feared self, self‐ambivalence, egodystonicity, early maladaptive schemas, and self concealment. Journal of Obsessive‐Compulsive and Related Disorders, 31, 100665. 10.1016/j.jocrd.2021.100665

[ab22061-bib-0029] Kelty, S. F. , Hall, G. , & Watt, B. D. (2011). You have to hit some people! measurement and criminogenic nature of violent sentiments in Australia. Psychiatry, Psychology and Law, 18(1), 15–32. 10.1080/13218710903566961

[ab22061-bib-0030] Lee, H. J. , & Kwon, S. M. (2003). Two different types of obsession: Autogenous obsessions and reactive obsessions. Behaviour Research and Therapy, 41(1), 11–29. 10.1016/S0005-7967(01)00101-2 12488117

[ab22061-bib-0031] Link, B. G. , Monahan, J. , Stueve, A. , & Cullen, F. T. (1999). Real in their consequences: A sociological approach to understanding the association between psychotic symptoms and violence. American Sociological Review, 64, 316–332. 10.2307/2657535

[ab22061-bib-0032] Lovibond, P. F. , & Lovibond, S. H. (1995). The structure of negative emotional states: Comparison of the depression anxiety stress scales (DASS) with the beck depression and anxiety inventories. Behaviour Research and Therapy, 33(3), 335–343. 10.1016/0005-7967(94)00075-U 7726811

[ab22061-bib-0033] Luciano, J. V. , Belloch, A. , Algarabel, S. , Tomás, J. M. , Morillo, C. , & Lucero, M. (2006). Confirmatory factor analysis of the white bear suppression inventory and the thought control questionnaire. European Journal of Psychological Assessment, 22(4), 250–258. 10.1027/1015-5759.22.4.250

[ab22061-bib-0034] Mills, J. F. , Kroner, D. G. , & Forth, A. E. (2002). Measures of criminal attitudes and associates (MCAA) development, factor structure, reliability, and validity. Assessment, 9(3), 240–253. 10.1177/1073191102009003003 12216781

[ab22061-bib-0035] Moulding, R. , Anglim, J. , Nedeljkovic, M. , Doron, G. , Kyrios, M. , & Ayalon, A. (2011). The obsessive beliefs questionnaire (OBQ): Examination in nonclinical samples and development of a short version. Assessment, 18(3), 357–374. 10.1177/1073191110376490 20634421

[ab22061-bib-0036] Moulding, R. , Coles, M. E. , Abramowitz, J. S. , Alcolado, G. M. , Alonso, P. , Belloch, A. , Bouvard, M. , Clark, D. A. , Doron, G. , Fernández‐Álvarez, H. , García‐Soriano, G. , Ghisi, M. , Gómez, B. , Inozu, M. , Radomsky, A. S. , Shams, G. , Sica, C. , Simos, G. , & Wong, W. (2014). Part 2. They scare because we care: The relationship between obsessive intrusive thoughts and appraisals and control strategies across 15 cities. Journal of Obsessive‐Compulsive and Related Disorders, 3(3), 280–291. 10.1016/j.jocrd.2014.02.006

[ab22061-bib-0037] Nagtegaal, M. H. , Rassin, E. , & Muris, P. (2006). Aggressive fantasies, thought control strategies, and their connection to aggressive behaviour. Personality and Individual Differences, 41(8), 1397–1407. 10.1016/j.paid.2006.05.009

[ab22061-bib-0038] OCCWG (2005). Psychometric validation of the obsessive belief questionnaire and interpretation of intrusions inventory—Part 2: Factor analyses and testing of a brief version. Behaviour Research and Therapy, 43(11), 1527–1542. 10.1016/j.brat.2004.07.010. 16299894

[ab22061-bib-0039] Pascual‐Vera, B. , Akin, B. , Belloch, A. , Bottesi, G. , Clark, D. A. , Doron, G. , Fernández‐Alvarez, H. , Ghisi, M. , Gómez, B. , Inozu, M. , Jiménez‐Ros, A. , Moulding, R. , Ruiz, M. A. , Shams, G. , & Sica, C. (2019). The cross‐cultural and transdiagnostic nature of unwanted mental intrusions. International Journal of Clinical and Health Psychology, 19(2), 85–96. 10.1016/j.ijchp.2019.02.005 31193125 PMC6517647

[ab22061-bib-0040] Pinto, A. , Greenberg, B. D. , Grados, M. A. , Bienvenu, O. J. , Samuels, J. F. , Murphy, D. L. , Hasler, G. , Stout, R. L. , Rauch, S. L. , Shugart, Y. Y. , Pauls, D. L. , Knowles, J. A. , Fyer, A. J. , McCracken, J. T. , Piacentini, J. , Wang, Y. , Willour, V. L. , Cullen, B. , Liang, K. Y. , … Nestadt, G. (2008). Further development of YBOCS dimensions in the OCD collaborative genetics study: Symptoms vs. categories. Psychiatry Research, 160(1), 83–93. 10.1016/j.psychres.2007.07.010 18514325 PMC2819420

[ab22061-bib-0041] Podubinski, T. , Lee, S. , Hollander, Y. , & Daffern, M. (2017). Patient characteristics associated with aggression in mental health units. Psychiatry Research, 250, 141–145. 10.1016/j.psychres.2017.01.078 28161609

[ab22061-bib-0042] Purdon, C. , & Clark, D. A. (1993). Obsessive intrusive thoughts in nonclinical subjects. Part I. Content and relation with depressive, anxious and obsessional symptoms. Behaviour Research and Therapy, 31(8), 713–720. 10.1016/0005-7967(93)90001-B 8257402

[ab22061-bib-0043] Purdon, C. , & Clark, D. A. (1994). Perceived control and appraisal of obsessional intrusive thoughts: A replication and extension. Behavioural and Cognitive Psychotherapy, 22(4), 269–285. 10.1017/S1352465800013163

[ab22061-bib-0044] Purdon, C. , Cripps, E. , Faull, M. , Joseph, S. , & Rowa, K. (2007). Development of a measure of egodystonicity. Journal of Cognitive Psychotherapy, 21(3), 198–216. 10.1891/088983907781494537

[ab22061-bib-0045] Rachman, S. (1997). A cognitive theory of obsessions. Behaviour Research and Therapy, 35(9), 793–802. 10.1016/S0005-7967(97)00040-5 9299799

[ab22061-bib-0046] Radomsky, A. S. , Alcolado, G. M. , Abramowitz, J. S. , Alonso, P. , Belloch, A. , Bouvard, M. , Clark, D. A. , Coles, M. E. , Doron, G. , Fernández‐Álvarez, H. , Garcia‐Soriano, G. , Ghisi, M. , Gomez, B. , Inozu, M. , Moulding, R. , Shams, G. , Sica, C. , Simos, G. , & Wong, W. (2014). Part 1—You can run but you can't hide: Intrusive thoughts on six continents. Journal of Obsessive‐Compulsive and Related Disorders, 3(3), 269–279. 10.1016/j.jocrd.2013.09.002

[ab22061-bib-0047] Rowa, K. , & Purdon, C. (2003). Why are certain intrusive thoughts more upsetting than others. Behavioural and Cognitive Psychotherapy, 31(1), 1–11. 10.1017/s1352465803001024

[ab22061-bib-0048] Rowell Huesmann, L. (1988). An information processing model for the development of aggression. Aggressive Behavior, 14(1), 13–24. 10.1002/1098-2337(1988)14:1<13::AID-AB2480140104>3.0.CO;2-J

[ab22061-bib-0049] Salkovskis, P. M. (1985). Obsessional‐compulsive problems: A cognitive‐behavioural analysis. Behaviour Research and Therapy, 23(5), 571–583. 10.1016/0005-7967(85)90105-6 4051930

[ab22061-bib-0050] Shafran, R. , & Rachman, S. (2004). Thought‐action fusion: A review. Journal of Behavior Therapy and Experimental Psychiatry, 35(2), 87–107. 10.1016/j.jbtep.2004.04.002 15210372

[ab22061-bib-0051] Sukhodolsky, D. G. , Golub, A. , & Cromwell, E. N. (2001). Development and validation of the anger rumination scale. Personality and Individual Differences, 31(5), 689–700. 10.1016/S0191-8869(00)00171-9

[ab22061-bib-0052] Veale, D. , Freeston, M. , Krebs, G. , Heyman, I. , & Salkovskis, P. (2009). Risk assessment and management in obsessive–compulsive disorder. Advances in Psychiatric Treatment, 15(5), 332–343. 10.1192/apt.bp.107.004705

[ab22061-bib-0053] Wheaton, M. G. , Abramowitz, J. S. , Berman, N. C. , Riemann, B. C. , & Hale, L. R. (2010). The relationship between obsessive beliefs and symptom dimensions in obsessive‐compulsive disorder. Behaviour Research and Therapy, 48(10), 949–954. 10.1016/j.brat.2010.05.027 20800750

